# Assessment of the Virulence of the *Burkholderia mallei* Strain BAC 86/19 in BALB/c Mice

**DOI:** 10.3390/microorganisms11102597

**Published:** 2023-10-20

**Authors:** Emanuelle Baldo Gaspar, Lenita Ramires dos Santos, Andréa Alves do Egito, Maria Goretti dos Santos, Cynthia Mantovani, Juliana da Silva Gomes Rieger, Guilherme Augusto de Sousa Abrantes, Paula Adas Pereira Suniga, Júlia de Mendonça Favacho, Ingrid Batista Pinto, Alessandra Figueiredo de Castro Nassar, Fernando Leandro dos Santos, Flábio Ribeiro de Araújo

**Affiliations:** 1 Embrapa South Livestock, BR-153, Km 632, 9 Vila Industrial, Rural Area, Mailbox 242, Bagé 96401-970, RS, Brazil; 2 Embrapa Beef Cattle, Rádio Maia Ave., 830, Campo Grande 79106-550, MS, Brazil; lenita.santos@embrapa.br (L.R.d.S.); andrea.egito@embrapa.br (A.A.d.E.); goretti.santos@embrapa.br (M.G.d.S.); flabio.araujo@embrapa.br (F.R.d.A.); 3 Embrapa Beef Cattle/Ministry of Agriculture, Livestock and Food Supply Scholarship, Embrapa Beef Cattle, Rádio Maia Ave., 830, Campo Grande 79106-550, MS, Brazil; cymant@hotmail.com (C.M.); juliana_vet11@hotmail.com (J.d.S.G.R.); guilherme.a.bio@hotmail.com (G.A.d.S.A.); ingridbatistabiotec@gmail.com (I.B.P.); 4 MAI/DAI Scholarship, Federal University of Mato Grosso do Sul, Cidade Universitária, Costa e Silva Ave., Campo Grande 79070-900, MS, Brazil; paula_adas@hotmail.com; 5 Postgraduate Program in Animal Science, Faculty of Veterinary Medicine and Animal Science-FAMEZ/UFMS, Federal University of Mato Grosso do Sul, Senador Filinto Muller Ave., 2443, Campo Grande 79074-460, MS, Brazil; 6 PIBIC/CNPq Scholarship, Embrapa Beef Cattle, Campo Grande 79106-550, MS, Brazil; juliamfavacho@gmail.com; 7 Center for Research and Development in Animal Health, Biological Institute, Conselheiro Rodrigues Alves Ave., 1252, São Paulo 04014-002, SP, Brazil; afcnassar@sp.gov.br; 8 UFPE Department of Veterinary Medicine, Federal Rural University of Pernambuco, Recife 52171-900, PE, Brazil; fernando.leandro@ufrpe.br

**Keywords:** glanders, BALB/c mice, pathogenicity, animal models

## Abstract

*Burkholderia mallei* is an aerobic, Gram-negative, non-motile bacillus. As an obligate mammalian pathogen, it primarily affects solipeds. Although rarely transmitted to humans, the disease it causes, glanders, is classified as a zoonosis. The bacterium was officially eradicated in Brazil in 1969; however, it reemerged after three decades. This study aims to assess the virulence of a specific *B. mallei* strain, isolated in Brazil, in BALB/c mice through intranasal infection. The strain, *B. mallei* BAC 86/19, was obtained from the tracheal secretion of a young mare displaying positive serology but no clinical signs of glanders. Post-mortem examinations revealed macroscopic lesions consistent with the disease, however. In mice, the LD_50_ was determined to be approximately 1.59 × 10^5^ colony-forming units (CFU)/animal. Mice exposed to either 0.1 × LD_50_ or 1 × LD_50_ displayed transient weight loss, which resolved after three or five days, respectively. *B. mallei* persisted within the liver and lung for five days post-infection and in the spleen for seven days. These findings underscore the detectable virulence of the Brazilian *B. mallei* BAC 86/19 strain in mice, which are relatively resilient hosts. This research points to the importance of the continued investigation of the virulence mechanisms and potential countermeasures associated with *B. mallei* infections, including their Brazilian isolates.

## 1. Introduction

*Burkholderia mallei*, the causative agent behind the disease known as glanders, presents itself as an aerobic, Gram-negative bacillus marked by its non-motile nature. As an intracellular facultative pathogen, it holds an obligate relationship with mammals [1]. Glanders primarily targets solipeds, and though it can sporadically be transmitted to humans from these animals, such cases are infrequent. Notably, the disease has a pronounced impact on various human occupations, including veterinarians, groomers, laboratory workers, butchers, and more, reflecting its occupationally hazardous nature [2]. Once diagnosed, the disease’s gravity in humans is stark, with untreated cases showing mortality rates reaching a staggering 95%. Even treated patients still face substantial mortality rates of up to 40%. This lethal potential has led the Centers for Disease Control and Prevention (CDC) to classify *B. mallei* as a category B bioterrorism agent [3]. Disturbingly, *B. mallei* has been historically employed as a biological weapon against animals, even in the 20th century, due to its poorly understood nature and its aerosol-transmissible characteristics [4].

Among equids, different susceptibility profiles exist, with donkeys typically experiencing an acute form, while horses exhibit resistance but are more prone to chronic manifestations [5]. Chronically infected horses act as the primary reservoir for *B. mallei* [6]. The disease progression is complex, featuring periods of illness intermingled with latent phases in horses with the chronic form [7]. Glanders affects the respiratory tract and skin, with nasal mucosa nodules that can result in distinct scarring after healing. Pulmonary and cutaneous manifestations are also common, contributing to the clinical picture [8].

When studying *B. mallei*, laboratory animals such as guinea pigs and hamsters are highly susceptible. At the same time, mice provide an intermediate level of susceptibility, making them valuable for vaccine development and immune function research [1].

Despite eradication efforts in many regions, glanders persists in various parts of the world, including Africa, Asia, the Middle East, and South America. In Brazil, despite successful eradication in 1969, a resurgence occurred in 1999 [9,10]. Diagnostic tools range from serological tests to bacterial isolation, with the latter being the gold standard [5]. Isolated bacteria must fulfill Koch’s postulates by inducing disease in animals. However, the virulence of *B. mallei* strains can vary widely, making an animal model imperative for assessing local isolates’ harmful potential and aiding vaccine development.

In fact, virulence depends on both the expression of virulence factors by the pathogen and the susceptibility of the host. Several virulence factors have already been described for *B. mallei* [11,12,13,14,15,16]. However, experiments demonstrating virulence in animal models with various strains of *B. mallei* isolated worldwide are scarce. Recently, we isolated *B. mallei* BAC 86/19 from a horse in Brazil, and there is currently no available information regarding the virulence of this strain in animal models.

Although *B. mallei* is a genetically homogeneous species, techniques with high discrimination power, such as SNP [17] or cgMLST [18] could distinguish genetic groups, and we postulate that these genetic differences can interfere with in vivo virulence.

Mice, specifically female BALB/c mice, are the ideal model for this study. Intranasal infection elucidates the virulence of a *B. mallei* strain sourced from a Brazilian horse. This model proves valuable due to the mice’s availability, adaptability to controlled research settings, and suitability for level-3 biosafety facilities, which are essential for handling *B. mallei*. Through this approach, this study contributes to a deeper understanding of the virulence profile of the aforementioned Brazilian *B. mallei* strain and its implications for local isolates and potential vaccine development.

## 2. Materials and Methods

### 2.1. Bacteria

*Burkholderia mallei* strain BAC 86/19 was isolated from a young mare with positive serology in the complement fixation screening test, and confirmed by Western blot. The mare was identified during a follow-up from the 2019 glanders outbreak in horses in Tatuí, São Paulo, Brazil, by the state veterinary service. The Western blot followed the National Equine Health Program’s guidelines. Tissue samples from the equid lungs, trachea, lymph nodes, heart, spleen, kidney, and liver, were collected during necropsy. At the Instituto Biológico, São Paulo, SP, Brazil, tissue samples were prepared via suspension in 0.85% sterile saline (at a ratio of 1:5 *w*/*v*). Subsequently, 10 µL of the prepared suspension was applied onto 5% sheep blood agar enriched with 1% glycerin and 2500 U of potassium benzylpenicillin. The plated samples were incubated at 37 °C for 48 to 72 h, after which morphological evaluation and Gram staining analyses were executed, as per established protocols [19,20,21]. Biochemical tests were performed, including catalase, oxidase, indole, nitrate reduction, Voges–Proskauer, motility, and sugar fermentation [19,20]. *B. mallei* confirmation involved PCR targeting IS407-*fliP*, generating a 528 bp fragment with specific primers (IS407-*fliP* F: 5′ TCAGGTTTGTATGTCGCTCGG 3′and IS407-*fliP* R: 5′ GCCCGACGAGCACCTGATT 3′) [22]. The reference *B. mallei* strain INCQS 00115 (ATCC 15310) from the Collection of Reference Microorganisms in Sanitary Surveillance, FIOCRUZ-INCQS, Rio de Janeiro, was used as a positive control, while sterile deionized water served as the negative control. The IS407-*fliP* PCR product was sequenced via Sanger methodology using BigDye^®^ Terminator v3.1 kit (Thermo Fisher Scientific, Vilnius, Lithuania) and analyzed using SeqScape^®^ Software v2.1 with sequence CP010348.1 from GeneBank as reference. Bacteria were maintained at −80 °C in BHI medium with 20% glycerol.

Aliquots from the bacterial stock were thawed at room temperature for the infection experiments. A 10% pre-inoculum in Luria–Bertani broth (LB) supplemented with 4% glycerol was incubated for 12–16 h at 37 °C. After growth, 1 mL aliquots were frozen in LB with 30% glycerol at −80 °C, for up to two months. Two days before infection, five aliquots were thawed, serially diluted tenfold and plated on blood agar supplemented with penicillin (500 U/mL) to estimate colony-forming units (CFUs)/mL. A new aliquot was diluted to the appropriate concentration, described below, to infect mice.

### 2.2. Mice Infection

Juvenile female BALB/c mice, aged 4 to 5 weeks, were acquired from the Mato Grosso do Sul Federal University in Campo Grande, MS, Brazil. The mice had a period of two weeks for acclimatization. During this time, they were housed within micro isolator cages, with pathogen-free conditions maintained, and had ad libitum access to rodent feed and water. These accommodations were situated within a controlled environment with a 12 h light cycle, located within the level-3 biosafety unit of Embrapa Beef Cattle in Campo Grande, MS, Brazil.

All experimental protocols underwent rigorous scrutiny and received approval from the Institutional Animal Care and Use Committee of Embrapa Beef Cattle (CEUA/CNPGC), as outlined in protocol number 007/2021. These procedures were meticulously conducted under the regulations stipulated by national animal welfare law.

The mice were subsequently randomized and distributed into five distinct groups, each consisting of five animals. For infection, the mice underwent anesthesia via intraperitoneal (i.p.) injection of ketamine (100 mg/kg) and xylazine (10 mg/kg), followed by intranasal (i.n.) infection of 5 µL volume per nostril. The anesthetized mice were positioned near an incandescent light source, providing thermal support to ensure complete recovery of the animals.

Safeguarding the well-being of the mice was a priority, with their ocular comfort maintained through regular application of carmellose sodium eye drops (5 mg/mL) using a piece of cotton. After the infection, daily monitoring of the animals ensued. Humane endpoints were established based on a scoring system previously described [23], with adjustments tailored to specific requirements. Criteria such as body condition, weight loss exceeding 20%, and signs of distress, contributed to the scoring. Cumulative scores reaching or exceeding 3 points led to the euthanasia of the affected animal. Additional indicators, namely vaginal discharge and hypothermia, were added into the grading system, increasing its sensitivity. Animals displaying significant discomfort were promptly and humanely euthanized using a threefold dosage of ketamine (300 mg/kg) and xylazine (30 mg/kg) compared to that used for anesthesia. The procedure culminated in complete exsanguination through cardiac puncture.

In the context of determining the lethal dose of 50% (LD_50_), the mice were subjected to challenges involving varying concentrations of *B. mallei* BAC 86/19 (7.4 × 10^2^, 7.4 × 10^3^, 7.4 × 10^4^, 7.4 × 10^5^, or 3.7 × 10^6^ CFU) i.n. Observations were sustained for 14 days, encompassing mortality, daily temperature measurements, and body weight fluctuations. Importantly, mice meeting humane endpoint criteria were included in the overall mortality assessment. The LD_50_ was subsequently estimated utilizing GraphPad Prism 6 software, employing non-linear regression with a fixed slope.

Following on from this, mice were exposed to challenges with 0.1 or 1 times the determined LD_50_ (1.59 × 10^4^ or 1.59 × 10^5^) and were humanely euthanized on post-infection days 1, 2, 3, 5, 7, and 14. The assessment also included bacteremia evaluations at 1, 2, 3, 4, 5, and 7 days post-infection (DPI), and daily temperature measurements and body weight recordings. The experiment sought to clarify how bacteria spread through the bloodstream and tissues and the impact of the infection on temperature and changes in body weight over time.

### 2.3. Bacteremia

Blood collection for bacteremia evaluation was performed through tip amputation, prioritizing minimal restraint to minimize distress [24]. Only 10 µL of blood was collected using an automated pipette with a sterile tip. The extracted blood was promptly placed on 5% blood agar, and enriched with penicillin (100 U/mL) and polymyxin (50 U/mL) to prevent undesired microbial growth. Plates were incubated at 37 °C for 48 h. Following the incubation phase, the CFUs were enumerated.

### 2.4. Determination of CFU in Tissues

After euthanasia, the lungs, spleen, and liver were placed separately in microtubes and weighed (the weight of the microtubes was accounted for). The organs were macerated with the plunger of sterile syringes in 1000 µL of sterile BHI media. The macerates were serially diluted 10 times in 96-well plates (micromethod) [25], followed by the plating of 10 µL of each dilution on blood agar with penicillin (100 U/mL) and polymyxin (50 U/mL). After drying the drops, plates were incubated for 48 h at 37 °C, and CFU was counted to estimate the CFU/g of organs.

### 2.5. Statistics

Bacteria doses were log-transformed (x = log(x)), and non-linear regression was fitted to mortality using a fixed slope of 1 to calculate the LD_50_. Kaplan–Meyer survival analysis was performed, and the log-rank test (Mantel–Cox) was applied. Continuous variables were expressed as mean (SD) and comparsofed by t-test when there were only two treatments or by one-way and two-way ANOVA when there were more than two treatments. After ANOVAs, Dunnett’s or Sidak’s multiple comparisons were applied. GraphPad Prism 6 (GraphPad by Dotmatics, San Diego, CA, USA) was used for the statistical analysis.

## 3. Results

Necropsy findings from the seropositive mare revealed a series of multiple miliary lesions within the lungs and liver. The trachea exhibited intense catarrhal discharge, while the mediastinal lymph nodes showed evident enlargement. In addition, splenic hypoplasia was evidenced (data undisclosed).

Among these samples, *B. mallei* was effectively isolated from a sample of tracheal secretion using a specialized medium, following the previously outlined methodology [22]. Colonies obtained this way exhibited small dimensions, assuming a gray and glossy appearance devoid of hemolytic activity. After phenotypic evaluation, the identification of the species was established via PCR and sequencing of the IS407-*fliP* DNA region.

This DNA sequence was subsequently deposited in GenBank, with the accession number MZ404924. A comprehensive comparative analysis exhibited flawless congruence, boasting 100% coverage and 100% identity with an array of *B. mallei* sequences present in GeneBank. Further examination revealed that this sequence retained coverage ranging from 96% to 99%, alongside identity spanning from 97.79% to 100%, when juxtaposed with other Brazilian isolates [26].

To validate a protocol to evaluate bacterial virulence in mice using the Brazilian *B. mallei* BAC 86/19 strain, female BALB/c mice were subjected to intranasal inoculation with varying dilutions. A stringent regimen of daily monitoring ensued, yielding a range of outcomes.

**Figure 1:**
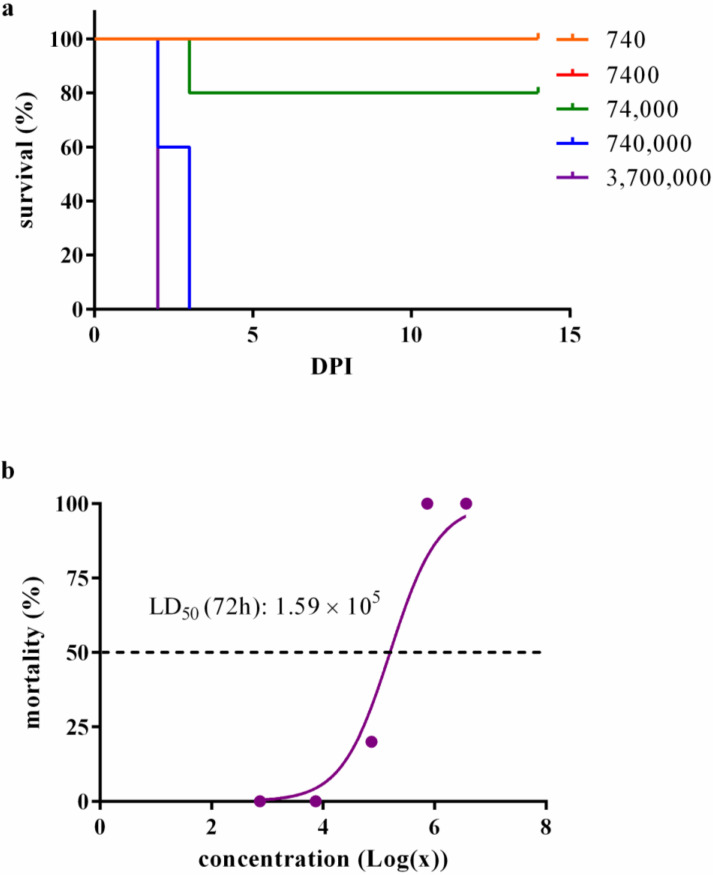
*Burkholderia mallei* BAC 86/19 virulence in BALB/c female mice. (**a**) Kaplan–Meyer survival analysis of mice challenged with five different *B. mallei* doses. BALB/c mice were challenged intranasally with five different doses of *B. mallei* BAC 86/19 (five animals per dose) and followed-up for 14 days. The red line (7400 CFU) is behind the orange one (740 CFU) because mortality was not observed in both groups. The log-rank (Mantel–Cox) test indicated the difference in the survival curves was *p* < 0.0001 at 14 DPI; (**b**) plot of the dose–response curve. The estimated LD_50_ was 1.59 × 10^5^ for the 14-day follow-up.

Following infection, a noteworthy pattern emerged: animals exposed to 3.7 × 10^6^ CFU and 40% (2/5) of those subjected to 7.4 × 10^5^ CFU required euthanasia 48 h post-infection. In a parallel vein, the remaining animals—3 out of 5 infected with 7.4 × 10^5^ CFU, and an individual infected with 7.4 × 10^4^ CFU—underwent euthanasia at the 72 h mark following infection (Figure 1a). Subsequent to this critical window, the experiment extended to the 14-day mark, during which no additional mortalities transpired, culminating in the experiment’s conclusion.

The calculated LD_50_ stood at 1.59 × 10^5^ up to the 14-day mark post-infection (DPI), a determination depicted in Figure 1b. Statistical scrutiny, namely the log-rank (Mantel–Cox) test, underscored a pronounced dissimilarity within the survival curves, signaling a *p*-value below 0.0001.

**Figure 2:**
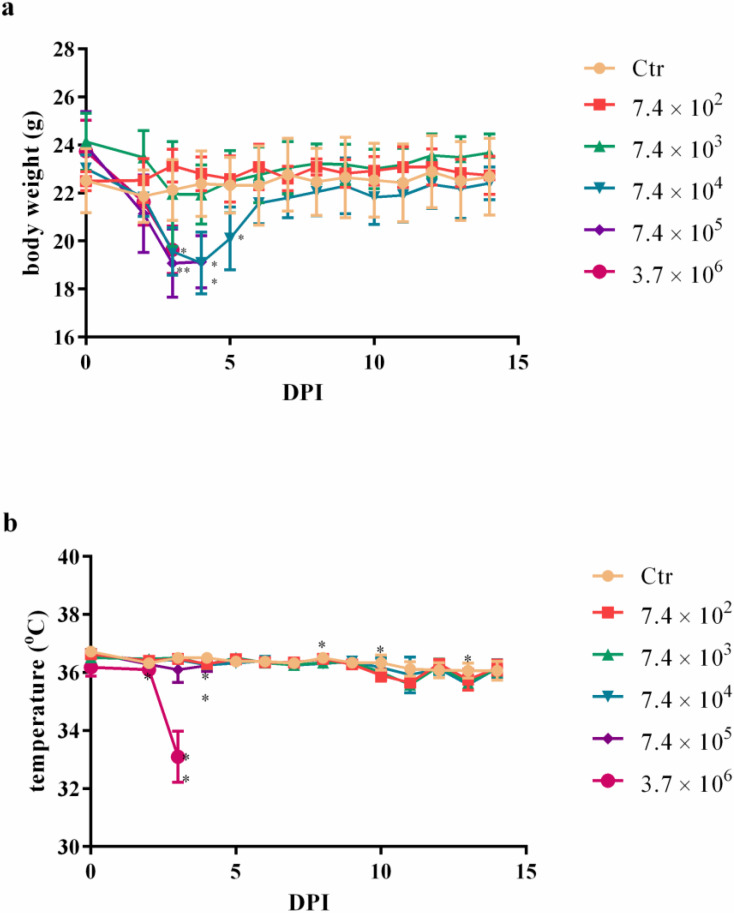
Body weight (**a**) and temperature (**b**) of mice challenged with different *Burkholderia mallei* BAC 86/19 doses. BALB/c female mice were challenged intranasally with five doses of *B. mallei* BAC 86/19 (five animals per dose) and followed up for 14 days (* *p* < 0.05; ** *p* < 0.01 in relation to the control).

A noteworthy subset of animals infected with 7.4 × 10^4^, 7.4 × 10^5^, and 3.7 × 10^6^ CFU displayed the emergence of purulent vaginal discharge, concurrently leading to the successful isolation of *B. mallei*. Notably, this trio of higher doses also exhibited a discernible decline in body weight commencing 48 h after infection. Notwithstanding, animals challenged with 7.4 × 10^4^ CFU survived, subsequently regaining lost body weight by the sixth DPI (Figure 2a). The cohort subjected to 3.6 × 10^6^ CFU encountered a significant drop in body temperature at the 48 h mark post-infection, precipitating mortality at this juncture (Figure 2b).

Subsequently, a new experiment was initiated, involving the intranasal administration of doses equivalent to either 0.1 or 1 times the previously determined LD_50_ (1.59 × 10^4^ and 1.59 × 10^5^ CFU/animal). Animals were monitored daily, including temperature and body weight records and physical examinations, aiming to observe human endpoint scores. Euthanasia was scheduled at intervals of 1, 2, 3, 5, 7, and 14 DPI to facilitate the quantification of *B. mallei* within the spleen, lung, and liver.

**Figure 3:**
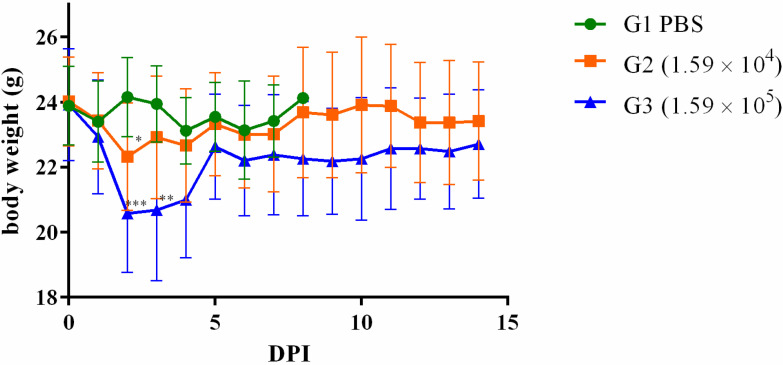
Body weight of animals infected with 0.1 or 1 × the LD_50_ of *Burkholderia mallei* BAC 86/19. BALB/c female mice were intranasally infected with 1.59 × 10^4^ (square; orange line) or 1.59 × 10^5^ (triangle; blue line) *B. mallei,* and body weight was measured daily from day zero up to 14 DPI (* *p* < 0.05; ** *p* < 0.01; *** *p* < 0.001).

**Figure 4:**
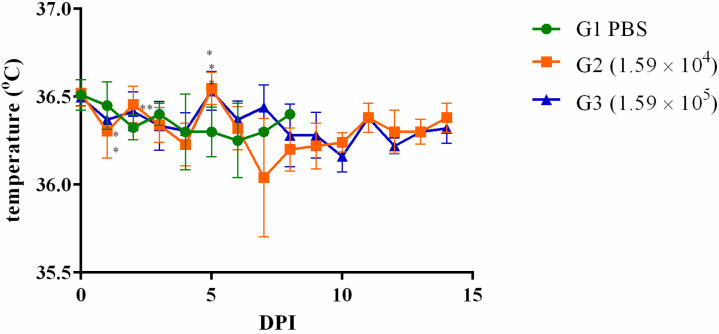
Body temperature of animals infected with 0.1 or 1 × the LD_50_ of *Burkholderia mallei* BAC 86/19. BALB/c female mice were intranasally infected with 1.59 × 10^4^ (square; orange line) or 1.59 × 10^5^ (triangle; blue line) *B. mallei*, and body weight was measured daily from day zero up to 14 DPI (* *p* < 0.05; ** *p* < 0.01).

Remarkably, as early as the second DPI, noticeable body weight reduction was observed across the animal subjects. In the cohort infected with 1.59 × 10^4^ CFU, the recovery of body weight commenced on the subsequent day (3 DPI). In contrast, animals challenged with 1.59 × 10^5^ CFU regained their body weight by the fifth DPI (Figure 3). However, the impact on body temperature exhibited a less consistent trend in response to infection with these dosages (Figure 4).

**Figure 5:**
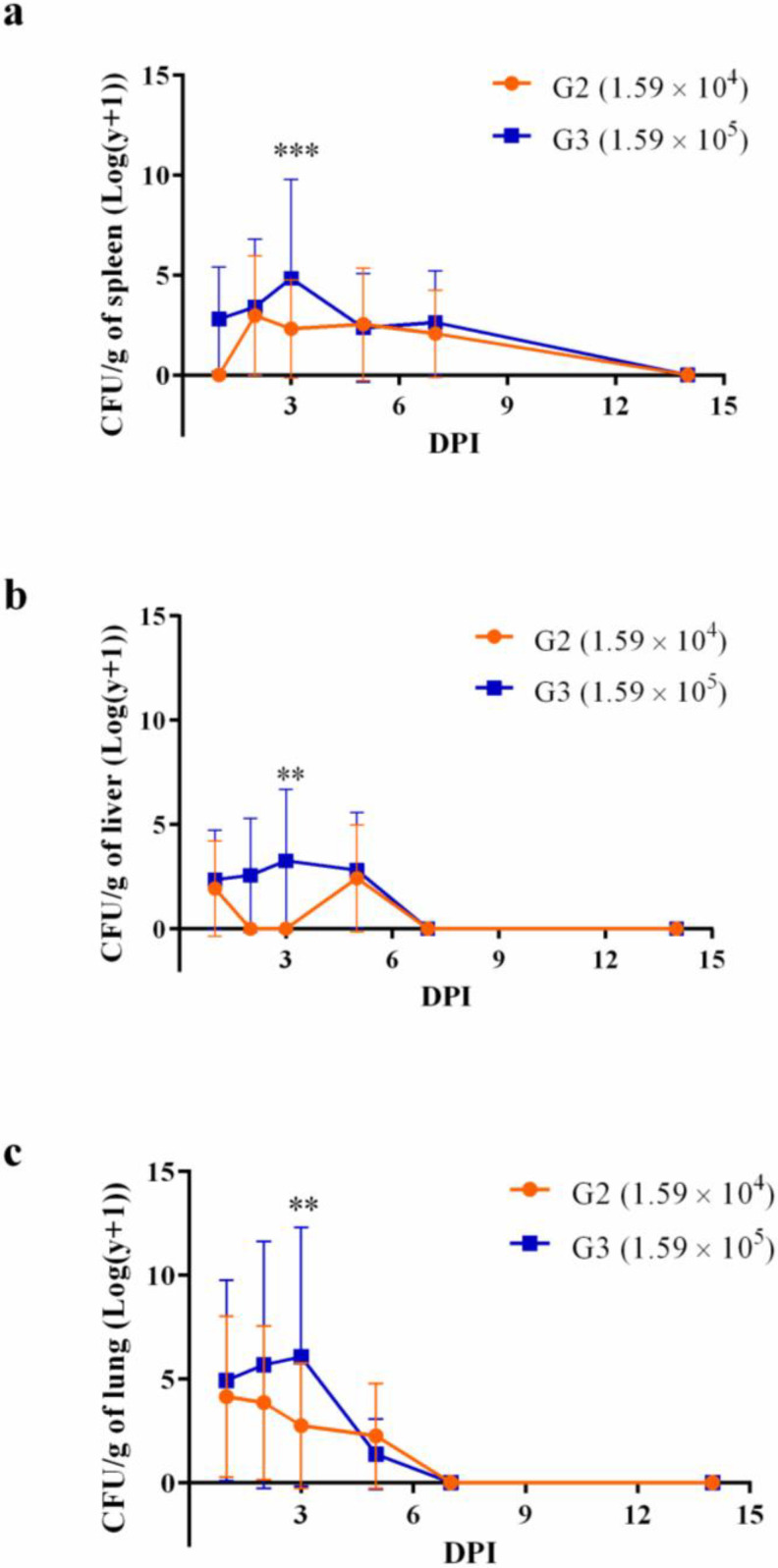
Bacterial burden in the spleen (**a**), liver (**b**), or lung (**c**) of animals infected with 0.1 or 1 × the LD_50_ of *Burkholderia mallei* BAC 86/19. BALB/c female mice were intranasally infected with 1.59 × 10^4^ (circle; orange line) or 1.59 × 10^5^ (square; blue line) *B. mallei* BAC 86/19 and euthanized on days 1, 2, 3, 5, 7, and 14 (five animals/time point) DPI for bacteria recovery from the spleen, liver, and lung (** *p* < 0.01; *** *p* < 0.001).

Following infection, the spread of *B. mallei* BAC 86/19 extended to the spleen, liver, and lung. Notably, animals administered the higher inoculum displayed\significantly elevated CFU/g of their tissue at the three DPI time points (Figure 5). The trajectory of bacterial persistence differed across organs, with the spleen harboring bacteria for at least seven days post-infection, while the lung and liver exhibited clearance as early as five days post-infection.

Bacteremia emerged as a transient occurrence, noted in one animal (20%) within each group at the 24 h mark post-infection. The bacterial load in the bloodstream reached 100 CFU/mL in the animals infected with 1.59 × 10^4^ CFU and 200 CFU/mL in the animals subjected to 1.59 × 10^5^ CFU. Subsequent time points, namely 48 and 72 h post-infection, witnessed a singular animal from the 1.59 × 10^5^ CFU group exhibiting bacteremia (100 CFU/mL).

## 4. Discussion

Glanders is an important infectious disease that, besides being a zoonosis, has a massive impact on the equine production chain due to the trade barriers imposed on endemic areas [27].

This study investigated the virulence characteristics of a specific strain of *B. mallei* from a horse in Brazil. Knowledge of the virulence of different *B. mallei* strains is crucial for assessing the potential risk to animal and human health. It can also help in understanding the modes of transmission and the potential for spreading glanders within animal populations and potentially to humans. This information is crucial for implementing control measures and quarantines to prevent outbreaks. Also, this information can be used to identify and track the emergence of more virulent strains, enhancing our ability to respond swiftly to outbreaks. Understanding the virulence of different strains can shed light on their ecological impact on animal populations and ecosystems. This knowledge can help in predicting disease dynamics and potential long-term effects.

The isolation of *B. mallei* BAC 86/19 occurred from a mare displaying macroscopic lesions suggestive of the disease during necropsy, despite the absence of clinical signs of glanders. This observation aligns with the known phenomenon that horses exhibit greater resistance to glanders than donkeys and mules and are predisposed to develop the chronic form of the disease. Distinguishing between disease forms can be challenging, particularly since the chronic variant is characterized by sporadic acute symptoms interspersed with apparent recovery and periods of latency, all while the infection persists [7]. However, isolating the bacterium from tissues or secretions remains a formidable endeavor.

Findings from this research highlighted the importance of employing a tissue/secretion bacterial culture and selection strategy. This approach has proven to enhance the sensitivity of *B. mallei* identification through PCR-specific gene amplification compared to conducting PCR directly from the lesion or secretion. This enhancement is likely attributed to the relatively low *B. mallei* load within the lesions [28].

Efforts were made to extend the comprehensive characterization of the isolated strain through biochemical analyses. Further validation was achieved via PCR and sequencing of the pertinent amplicon, culminating in a full genome sequencing procedure (GenBank accession number JANCTE000000000.1), which will allow future typing at the strain level to compare this isolate with others worldwide distributed [17,18].

In solipeds, *B. mallei* can be transmitted by ingesting contaminated food and water, penetration through abrasions on the skin or mucous membranes, or even exposure to aerosol forms [8]. The route of infection can be determinant in infection models.

At first, the peritoneal route was used to infect Swiss mice with 10^5^, 10^6^, or 10^7^ colony-forming units (CFU)/animal. However, the animals were followed up to 45 days post-infection (DPI), and no clinical signs were observed. Animals were euthanized 4 or 45 DPI, and no bacteria were recovered from the liver, lung, and spleen. Remarkably, the challenge of 10^7^ CFU of *B. mallei* BAC 86/19 was the equivalent of the lethal dose described in the existing literature pertaining to the mice’s intraperitoneal model [29]. Despite this, this dose proved inadequate in inducing disease manifestation within the context of the experiment. This intriguing observation underscores the intricate interplay of factors that influence the pathogenesis of *B. mallei* within this specific experimental setting.

Since glanders is primarily a respiratory or cutaneous disease [7], a switch was decided, thus altering the route of infection. The aerosol infection model is feasible [23,30] but can represent a risk to laboratory workers, as cases of human glanders have been related to this occupation [31]. Moreover, it depends on using specific apparatus, and depending on the equipment and technique used to generate the aerosol, it could be hard to generate a previous quantification of the inoculated CFU. Oropharyngeal aspiration was also used as a model of infection with *B. mallei* and *B. pseudomallei*, but it requires trained staff to perform the procedure [32]. For the reasons discussed above, the intranasal model of infection was chosen since it delivers the inoculum to the respiratory airway and has been used to infect mice with *B. mallei* as a respiratory infection model [33,34,35].

First, the mortality rate after i.n. infection was documented with five different bacterial doses, and we estimated that the LD_50_ was 1.59 × 10^5^ for 72 h up to 14 DPI. Others have reported LD_50_ ranging from 251 to 10^4^ after aerosol infection [16,36,37,38,39]; 1.7 × 10^3^ after oropharyngeal infection [32]; or from 6.81 × 10^2^ to 8 × 10^4^ CFU after intranasal infection [33,34,35,40,41]. Virulence depends on factors from both the pathogen and the host. BALB/c mice are more resistant than hamsters to *B. mallei* infection through the intraperitoneal route [29,42]. The closely related species *B. pseudomallei* is more virulent to BALB/c than to C57BL/6 mice and to older animals than their younger counterparts. Regarding sex, no significant difference in susceptibility was reported [43]. The infection route and bacterial strain is also key to the median lethal dose for *B. pseudomallei* [44]. However, in this 14-day follow-up, all the mortality was observed in the first 3 DPI. This acute mortality has also been demonstrated by others [11,33,34,35,41,45]. Regarding *B. mallei*, these differences in virulence can be explained by the different expressions of virulence factors. Type III secretion system effector bopA [45]; inner membrane energy transfer protein TonB [12]; capsule; LPS [13]; flagella [14]; and type VI secretion system [15] are some examples of virulence factors described for *B. mallei*. Moreover, two putative proteins related to ubiquitination, phagosomal escape, and host actin-cytoskeleton rearrangement processes, as well as a putative phosphatase, were demonstrated to be virulence factors [16]. Virulence factors interfere with important cellular processes in the hosts, for example, cell signaling, cytoskeleton rearrangements, autophagy suppression, or apoptotic control [16].

Other animals were challenged with 0.1 or 1 × the LD_50_. It was possible to note a body weight drop in the days following the infection with posterior recovery. No deaths were observed during this second phase of the experiments, and animals were euthanized 1, 2, 3, 5, 7, or 14 DPI. Bacteria were recovered from the spleen, liver, and lung but persisted longer in the spleen. Although the bacterial load in the lungs was slightly higher than the spleen and liver in 1, 2, and 3 DPI, it dramatically decreased on day 5 and was absent from 7 DPI. These results corroborate previous ones, in which animals euthanized on days 2 [35] or 10 [45] presented a higher bacterial load in the lungs compared to the spleen or liver. But, when euthanasia occurred 21 [34], 34 [11], or 35 [33] days post-infection, the number of bacteria was higher in the spleen compared to the lungs. It is not surprising that the mice showed clearance of the bacteria in the organs and recovered from the disease since sublethal doses of bacteria [29] were used, and mice are considered intermediate in their susceptibility to the disease [1]. Thus, they are considered ideal models for vaccine development or immune functional studies [1]. As far as is known, this is the first time that a *B. mallei* strain isolated in Brazil was shown to be virulent to mice.

Altogether, the results obtained showed the detectable virulence of the Brazilian *B. mallei* BAC 86/19 strain in BALB/c mice, which belong to an animal species relatively resistant to glanders, and showed that these animals could be a good model to study future strains isolated in Brazil, as well as evaluate possible treatments or vaccines.

## Data Availability

The datasets used and/or analyzed during the current study are available from the corresponding author upon reasonable request.
